# Supplementary material for: Physicians and Machine-Learning Algorithm Performance in Predicting Left-Ventricular Systolic Dysfunction from a Standard 12-Lead-Electrocardiogram
Source: J Clin Med. 2022 Nov 15;11(22):6767. doi: 10.3390/jcm11226767 (PMC9699306; doi:10.3390/jcm11226767)
Supplement: Supplementary file 1 [file jcm-11-06767-s001.zip › jcm-1936414-supplementary.pdf]

Supplementary Materials

Table S1. Baseline characteristics of patients with ejection fraction EF ≤ 35% and those with EF > 35%.

Index	EF > 35% (*n* = 11,787)	EF ≤ 35% (*n* = 2033)	*p*-Value
Age (median years [IQR])	69.8 [59.3–80.3]	70.6 [61.5–80.2]	0.002
Male (*n*; %)	6962; 59.1%	1514; 74.5%	<0.001
Family history of coronary artery disease (*n*; %)	2556; 23.9%	849; 43.1%	<0.001
Diabetes Mellitus (*n*; %)	5584; 52.2%	1353; 68.8%	<0.001
Hypertension (*n*; %)	8012; 75.0%	1615; 82.1%	<0.001
Chronic kidney disease (*n*; %)	3476; 32.5%	1066; 54.2%	<0.001
Dialysis (*n*; %)	2434; 22.8%	842; 42.8%	<0.001
Peripheral vascular disease (*n*; %)	2796; 26.2%	899; 45.7%	<0.001
Hyperlipidemia (*n*; %)	6613; 61.9%	1414; 71.8%	<0.001
Obesity (*n*; %)	3741; 35.0%	967; 49.6%	<0.001
Atrial fibrillation/flutter (*n*; %)	3756; 35.1%	1050; 53.4%	<0.001
Pacemaker (*n*; %)	2527; 23.6%	905; 46.0%	<0.001
Chronic obstructive pulmonary disease (*n*; %)	3033; 28.4%	923; 46.9%	<0.001
Smoking history	4552; 42.6%	1218; 61.9%	<0.001
Stroke/Transient ischemia accident (*n*; %)	2972; 27.8%	928; 47.2%	<0.001
Ischemic heart disease	4627; 43.3%	1344; 68.3%	<0.001
Coronary artery bypass surgery (*n*; %)	2684; 25.1%	924; 47.0%	<0.001
Percutaneous coronary intervention (*n*; %)	2858; 26.7%	948; 48.2%	<0.001
Ejection fraction (median % [IQR])	55 [45–60]	30 [25–35]	<0.001

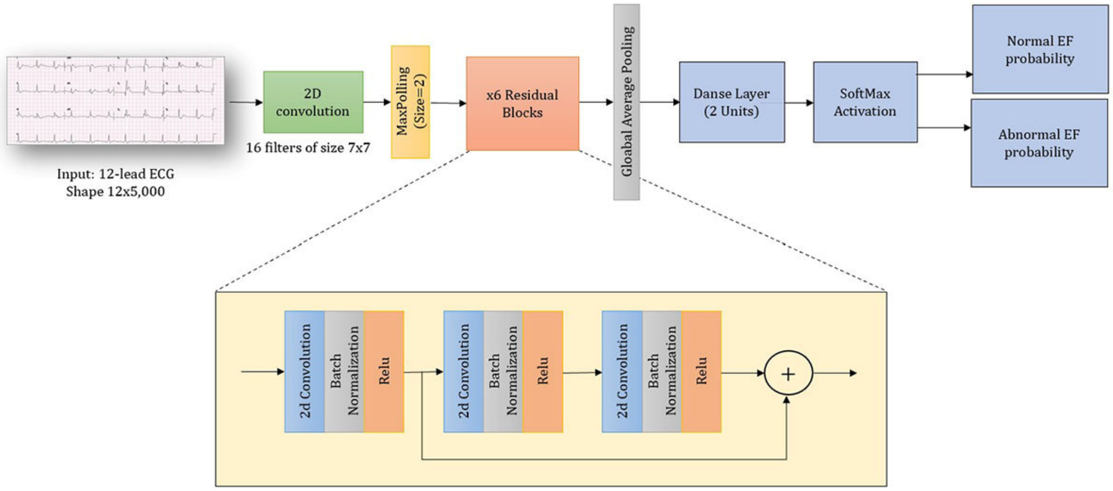

Figure S1. Residual Network architecture diagram.
